# The Development of a Bacterial Nanocellulose/Cationic Starch Hydrogel for the Production of Sustainable 3D-Printed Packaging Foils

**DOI:** 10.3390/polym16111527

**Published:** 2024-05-29

**Authors:** Špela Dermol, Bojan Borin, Diana Gregor-Svetec, Lidija Slemenik Perše, Gregor Lavrič

**Affiliations:** 1 Faculty of Natural Sciences and Engineering, University of Ljubljana, Aškerčeva cesta 12, 1000 Ljubljana, Slovenia; spela@dermol.si; 2 Pulp and Paper Institute, Bogišićeva ulica 8, 1000 Ljubljana, Slovenia; bojan.borin@icp-lj.si; 3 Faculty of Mechanical Engineering, University of Ljubljana, Aškerčeva cesta 6, 1000 Ljubljana, Slovenia; lidija.slemenik.perse@fs.uni-lj.si

**Keywords:** 3D printing, bacterial nanocellulose, hydrogel, foil, packaging

## Abstract

Polymers have become an important part of everyday life, but most of the polymers currently used are petroleum-based. This poses an environmental problem, especially with respect to products that are quickly discarded. For this reason, current packaging development focuses on sustainable materials as an alternative to synthetic ones. Nanocellulose, a relatively new material derived from cellulose, has unique properties such as high strength, low density, high surface area, and good barrier properties, making it popular in various applications. Additionally, 3D printing technologies have become an important part of industrial and commercial processes, enabling the realization of innovative ideas and functionalities. The main aim of this research was to develop a hydrogel of bacterial nanocellulose with suitable rheological properties for the 3D printing of polymer foils. Three variations of bacterial nanocellulose hydrogel differing in ratios of bacterial nanocellulose to cationic starch were produced. The rheological studies confirmed the suitability of the hydrogels for 3D printing. Foils were successfully 3D-printed using a modified 3D printer. The physical-mechanical, surface, and optical properties of the foils were determined. All foils were homogeneous with adequate mechanical properties. The 3D-printed foils with the highest amount of cationic starch were the most homogeneous and transparent and, despite their rigidity, very strong. All foils were semi-transparent, had a non-glossy surface, and retained poor water wettability.

## 1. Introduction

Polymers are an important part of today’s everyday life because of their low cost, wide range of applications, properties, and ease of production. In 2018, 359 million tons of plastic were produced worldwide, comprising 51% from Asia, 30% from China, 17% from Europe, 7% from the Middle East and Africa, and the rest from other parts of the world [1]. One of the most critical and problematic aspects of plastic packaging is that most of the polymers currently used and produced are petroleum-based. These materials are non-degradable, difficult to dispose of and recycle, and raise issues concerning the depletion of oil resources and environmental controversy. As consumers have become more eco-conscious, packaging development focuses on sustainable materials as alternatives to synthetics [2]. Cellulose, among other natural polymers, can serve as a relatively inexpensive, non-toxic substance, making it a versatile component in various durable materials and applications. It is available worldwide, with an estimated annual global production of around 75 to 100 billion tons [3,4,5]. Nanotechnology and fundamental technological advances now offer further opportunities to improve the properties of such materials [6]. The European Commission defines nanomaterials as substances with 50% or more of the particles having one of the outer dimensions in the range 1 nm–100 nm [7].

Nanocellulose has several favorable properties. These include high strength, low density, high specific area, transparency when processed into thin films, biodegradability, etc. Such properties have put nanocellulose at the center of a rapidly developing field of research [8,9]. Nanocellulose can be divided into three basic categories [10]: Cellulose Nanocrystals (CNC), Cellulose Nanofibers (CNF), and Bacterial Nanocellulose (BNC). Due to the different sources and extraction techniques, some engineered nanocellulose products differ in shape, particle size, and degree of crystallinity [5].

The main focus of this research was on the development of hydrogels for 3D printing based on BNC. BNC differs from CNC and CNF in structure, properties, and origin. Due to its unique physico-chemical and biological properties, which include high strength, biocompatibility, biodegradability, and purity, it is becoming increasingly researched in various fields [11]. It can be produced using several bacterial genera, such as *Acetobacterium xylium*. The bacteria produce a cellulose polymer gel composed of finely structured cellulose fibrils ranging from 20 to 100 nm wide, with a basic nanofibril structure of about 2–4 nm in diameter [12]. The gel is unique and pure in its chemical structure as it does not contain other components or functional groups, such as lignin and hemicellulose [9,12]. Its many positive properties, such as mechanical strength, high absorption capacity, porosity, flexibility, and biodegradability, some of which are superior to those of CNC and CNF, make it suitable for many different applications. It is, therefore, not surprising that it has been receiving increasing attention in recent years [11]. Some applications include improving physical and mechanical properties in paper production, use in medicine as a drug delivery system, and use in cosmetics as masks, skin repair aids, etc. From an ecological point of view, bacterial nanocellulose is very important, as it can be obtained from a by-product such as the mother of vinegar resulting from standard vinegar production [12,13,14]. In addition, producing BNC by extracting and purifying the pulp does not require intensive chemical treatments [15]. Although its beneficial characteristics are numerous, its production faces difficulties in terms of high capital requirements, production costs, and scaling up of BNC biosynthesis, resulting in poor BNC production on a commercial scale [11,16].

In addition to BNC, starch can also be used to prepare the hydrogels. Starch is a polysaccharide that is composed of amorphous amylose and crystalline amylopectin. Both consist of glucose units [17,18]. Starch granules are deposited in the plastids of higher plants and vary in size and shape depending on the botanical source. Starch is a popular industrial commodity due to its high availability, low cost, renewability, and ease of changing structure. Most commercially available starches come from the fruits of maize, rice, and wheat, as well as potato tubers and roots such as cassava [18,19]. Maize is estimated to account for 83% of the world’s total starch production, and the majority, around 60% of all starch, is used in the food industry. Starch granules usually contain other chemical compounds such as proteins, lipids, and minerals [19,20].

Cationic starch is one form of modified starch. It is formed by the insertion of a cationic part, i.e., cationic ether groups such as amine, ammonium, sulphonium, etc., into the basic structure of the starch [21,22]. Its production involves treating a suspension of partially swollen starch granules with a reactive chemical such as epoxypropyl trimethylammonium chloride. The active site of the starch molecule is usually C6, where the reagent typically binds to the starch. The result is a positively charged starch, usually presented in dry powder form [13]. It is used for various functionalities such as emulsification and dry strength, as a flocculating agent [22], and in addition to negatively charged cellulose, paper, textiles, cosmetics, and others [23].

Fused deposition modeling (FDM), a type of material extrusion technique used in 3D printing, is considered one of the more widespread and frequently used 3D printing techniques. Its success is based primarily on using non-toxic, user-friendly, and cost-effective materials [24]. The FDM 3D printing technique consists of a simple process. First, a filament composed of different materials, usually thermoplastics and composites such as Acrylonitrile Butadiene Styrene [25], Polylactic Acid (PLA) [26], and Polypropylene (PP) [27], is heated and melted to a semi-fluid consistency, and then a nozzle extrudes the material in a thin layer. The process is then repeated, building up the layers and the shape of the 3D-printed object until all the layers of the 3D object are printed according to the computer-aided design file, its G-code, and the 3D printer’s source software [24,28].

FDM technology is used in the 3D printing of biofibers, including CNF-based and CNC-based biofibers. Direct Ink Writing (DIW) is another technique used in 3D printing with materials such as polymers and hydrogels. Most of the research reported used this technique for 3D printing hydrogels containing nanocellulose.

Tang et al. [29] created nanocellulose hydrogel materials with an added photosensitive component. In addition, they have demonstrated the feasibility of 3D printing such hydrogels through solidification and the production of UV-polymerized nanocellulose-based objects. Ambone et al. [30] investigated the effect that small amounts of CNF can have on PLA filaments. They found that the tensile properties of 3D-printed composite objects are greatly improved with as little as 1% nanocellulose in the dry matter.

A study by Xu et al. [31] investigated the 3D printing of porous gelatin and CNC-oxidized nanocellulose supports used in tissue engineering. The analysis was carried out using several hydrogels with different ratios of CNC and gelatine, and their behavior was investigated at various temperatures, from 4 to 30 °C. The first part of the study, the cross-linking, was carried out at different temperatures and lengths. The second part, 3D printing, was first performed by designing a pneumatic extrusion experiment to determine the flow rate and pressure required to 3D print the objects. The 3D printing was followed by cooling and drying of the 3D-printed structures. They found that the addition of oxidized nanocellulose improved both the rheological and mechanical properties of the samples and concluded that the process was acceptable and highly efficient.

Han et al. [32] performed a study of CNF and alginate/gelatin materials for FDM 3D printing. The rheological, morphological, mechanical, and other properties of five samples with different concentrations of CNF, i.e., 0%, 0.25%, 0.5%, 0.75%, and 1%, were observed. Their findings showed that adding CNF to alginate and gelatine bio-inks generally improves the structural and mechanical properties but negatively impacts the elongation at break and the compression rate of the hydrogels. Another study [33] showed the feasibility of 3D printing all wood-based bio-based materials, such as CNF, mixed with xylan, a source of hemicellulose. The addition of xylan further improved the stability and resulted in cross-linking of the 3D-printed object. Leppiniemi et al. [34] studied the behavior of CNF samples made in different ratios with alginate and glycerin. Their research showed that such materials are a good option for 3D printing. With the addition of CNF, stability increased. The addition of glycerin resulted in the stability of the printed object at room temperature but also led to the need to freeze-dry such objects. The study showed that an object 3D-printed with pure CNF collapsed due to water evaporation. Even after freezing, the items remained brittle and inelastic. Another study used CNF hydrogel with added alginate to 3D print human cartilage tissues. Markstedt et al. [35] produced hydrogels with different proportions of the two components and 3D-printed them using FDM technology. They found that a sample with 80% CNF and 20% alginate was optimal for their purpose. Olmos-Juste et al. [36] 3D-printed CNF/alginate hydrogel patches. The results of their study showed them that hydrogels with less than 3% dry matter by weight of CNF were unsuitable for 3D printing and did not retain their shape after printing.

Hydrogels used in extrusion-based 3D printing are mostly printed by the 3D bioprinting method. Three-dimensional bioprinting refers to the 3D printing of structures using bioinks consisting of biomaterials to be fabricated, living cells, and essential nutrients [37]. BNC is often used in addition to other bio-inks to modulate their properties, not as a main component. Also, BNC is limited in 3D printing because of the complex protofibrous structure that affects the solution viscosity and causes it to frequently clog the nozzles, which restrains the use of the FDM printing technique [38].

As mentioned above, the number of studies on the use of BNCs is small, but Wu and colleagues [39] have nonetheless studied 3D-printed objects enriched with growth cells for the treatment of nerve injuries. An alginate/gelatin methacrylate/BNC hydrogel was produced in different ratios. BNC was used in minimal quantities, i.e., 0.3%, to improve mechanical strength. Huang et al. [40] used bacterial nanocellulose in their study to improve the properties of a silk fibroin/gelatin hydrogel object. They found that the addition of BNC drastically improved the tensile strength of 3D-printed objects. Different ratios of BNC were used, i.e., 0.35 wt%, 0.70 wt%, and 1.40 wt%, and these were added to precise proportions of silk fibroin, gelatine, and glycerol. Apelgren et al. [41] designed a bio-based material for in vivo cartilage formation in their study. They used a special method to disassemble the BNC fibers, the aqueous counter-collision method, and hydrolysis. This resulted in longer BNC fibers, fewer negative charges, and excellent mechanical and structural properties after 3D printing.

From the literature [37,38,42,43,44], it is evident that hydrogels in 3D bioprinting using nanocellulose are mainly used for tissue engineering applications. In our research, we were interested in fabricating a 3D-printed structure from BNC-based hydrogels using the FDM technique for packaging applications.

The research was conducted in three phases. The first phase involved research and preparation of the hydrogels, followed by the modification of the 3D printer as described by Bessler et al. [45], with additional adjustments and upgrades. The 3D printer was thus built with fewer additional particles needed; the 3D printing itself did not involve modifying and integrating additional codes in the software and was, therefore, suitable for 3D printing the hydrogel in a short time. An analysis of the physical–mechanical and optical properties of the 3D-printed foils followed.

## 2. Materials and Methods

The bacterial nanocellulose was obtained using the process described by Lavrič et al. [46] from the mother of vinegar. The mother of vinegar was produced using the classical (static) process of apple vinegar production. The BNC was washed and cut into rounds, heated to 40 °C, continuously stirred for 40 min in 50% ethanol (Pharmachem), rinsed, and again heated and stirred at 40 °C in 0.1 M NaOH for 40 min. The mixture was then homogenized (Kinematica Polytron PT45/80) for 1 min at 7000 revolutions per minute (RPM). Additional treatment in NaOH under the same conditions was applied again, further reducing the number of impurities in the BNC sample after the treated sample was filtered and continuously filtered, resulting in a thin plate of BNC [12,46].

The second polymer chosen was the cationic starch PAPIRAN SKM-42 from the Slovenian manufacturer Helios Kemostik (Domžale, Slovenia). According to the manufacturer, this product is a medium-DS cationic starch containing quaternary ammonium functional groups and is not soluble in cold water. The degree of substitution (DS) of cationic starch is 0.039–0.045, as reported by the manufacturer. Product information is presented in Supplementary Materials.

### 2.1. Laboratory Production of Mixtures of Hydrogel and Cationic Starch

**Homogenization of the BNC**

The BNC suspension in water with a concentration of about 1%, prepared following the method of Lavrič et al. [12], was treated for 12 min at 7000 RPM with a homogenizer (Polytron PT 45/80, Kinematica, Switzerland), which homogenized the mixture and allowed the BNC fibrils to separate.

**Ultrasonic probe treatment**

The sample, treated with a homogenizer, was placed in an ice bath in small 200 mL beakers, and an ultrasound probe (Vibracell VCX 750, Sonics, Oklahoma, OK, USA) was inserted. The sample was treated in three intervals, each consisting of a 10 min ultrasonic treatment of 80% amplitude at 700 W, with the sample being treated periodically every 30 s. Between each 20 min interval, the sample was stirred to distribute the heat generated during the treatment more uniformly, and ice was added to the bath around the beaker if necessary. The temperature increase was allowed to reach a maximum of 40 °C.

**Calculation of dry matter in the sample**

The treated samples were filtered to remove most of the water. A larger Büchner funnel and filter paper were used, and the total mass of the sample was distributed over several filter papers to ensure uniform filtration and to avoid filling the pores of the filter paper, which would have resulted in uneven filtration and a much slower process. The total mass of all the filtrates was then combined and mixed, resulting in a homogeneous final sample. The dry matter content was then calculated according to the standardized procedure (ISO 638-1:2022) [47]. A small amount of the sample (the size of a fingernail) was placed in a weighing bottle and weighed; the bottle with the lid had previously been weighed without the contents. The sample was dried at 105 °C for 1 h and then transferred to a desiccator, where it stood for 5 min. After weighing the sample and recording the results, drying was repeated for 15 min at the same temperature, followed by 5 min in a desiccator. Then, the sample was finally weighed, and the deviation of the results was calculated. The work was completed when the mass was no longer changing.

**Preparation of composite hydrogel**

Calculations of the amount of cationic starch required were performed according to the variations of the final hydrogel. All samples were based on 2% dry matter in the final weight, and three samples were analyzed, as shown in Table 1.

According to the calculations using equations 1 and 2, the cationic starch was then prepared in a water bath. An example of the calculation can be seen in Supplementary Materials Table S1. The appropriate mass of starch was weighed, tap water was added, and the mixture was heated at 80 °C for 1 h with a magnetic stirrer, subsequently cooled to room temperature, and then mixed with the previously determined mass of bacterial nanocellulose. Samples were made up to the final mixture just before 3D printing to ensure freshness and the best printing and sample conditions.

(1) $$W_{a}=\frac{B_{p}}{B}\left(B\left(50+\frac{49}{R}\right)-1\right)$$

(2) $$S=\frac{B_{p}}{R}$$

W_a_ … final mass of water in the mixture [g]

B_p_ … BNC dry weight [g]

B … initial mass of sample; BNC + water [g]

R … BNC/cationic starch ratio [/]

S … added cationic starch amount [g]

A sample of pure nanocellulose was prepared as a comparison sample, but because of non-homogeneity and separated components, i.e., fibrils, it could not be 3D-printed and was excluded from this study. Our interest was in obtaining hydrogels with the prevailing content of nanocellulose. At this stage, we were not interested in hydrogels with a prevailing cationic starch content. We opted for nanocellulose due to its derivation from an alternative raw material source (mother vinegar), aligning with sustainability principles. Additionally, our focus on nanocellulose hydrogels was driven by the relatively unexplored nature of this material in hydrogel preparation, presenting an opportunity for innovative research.

### 2.2. Analysis of BNC

#### 2.2.1. X-Ray Diffraction (XRD) Analysis

XRD is a non-destructive analytical method specifically designed for qualitative and quantitative phase analyses of crystalline materials. In this study, the XRD system utilized was the Empyrean PANalytical X-ray diffractometer (Malvern Panalytical Ltd., Malvern, UK), equipped with a high-resolution goniometer and a ceramic X-ray tube with a copper anode. The measurement was conducted in a theta/theta configuration with a circle diameter of 480 mm, allowing for precise angular measurements within an angular range of −110° < 2θ < 168°. The XRD diffractogram obtained from the analysis provided information on the position, size, and shape of diffraction peaks, as well as the characteristics of the crystalline phases present in the sample. The degree of crystallinity was then calculated in accordance with the so-called Segal’s method [48].

#### 2.2.2. Zeta Potential Analysis

The zeta potential was determined using the Mütek™ SZP-10 System (BTG Instruments, Eclepens, Switzerland). This system is commonly employed to measure the surface charges of solid materials in aqueous systems. Prior to analysis, BNC was suspended in water. The concentration was set to 1%.

#### 2.2.3. Fourier Transform Infrared Spectroscopy (FT-IR)

FT-IR measurements were conducted using the spectrometer Spectrum Two (Perkin Elmer, Waltham, WA, USA) with a LiTaO_3_ detector in an Attenuated Total Reflectance (ATR) mode. The sample collection was obtained using 16 scans, in the range of 4000 to 400 cm^−1^, at a resolution of 4 cm^−1^. The samples underwent no special pre-treatment before measurement, maintaining their original properties.

### 2.3. Analysis of Hydrogels on the Rheometer

The rheometric measurements were conducted at the Faculty of Mechanical Engineering, University of Ljubljana. All tests were performed using an MCR302 rheometer (Anton Paar, Graz, Austria) with two-sandblasted plate geometry (PP-25/s). All measurements were carried out at a constant temperature of 25 °C and in a covered area of the rheometer to prevent the sample from drying out and to ensure a constant temperature during the measurements. Amplitude tests were performed for all samples in oscillatory mode and involved increasing the shear stress from τ = 0.1 to 1000 Pa at a constant oscillation frequency of f = 1 Hz. Amplitude tests were used to determine each sample’s linear viscoelastic range (LVR) separately. The maximum LVR stress for all samples was determined at τ = 2–8 Pa, depending on the sample. Frequency tests were performed at constant stress within a linear viscoelastic range and with different values of the oscillation frequency from f = 100 to 0.1 Hz.

### 2.4. 3D printing on a Customized Prusa i3 MKS3+ 3D Printer

Two objects were designed for 3D printing, i.e., 5 × 5 cm and a 7 × 1.5 cm plate. The dimensions of these objects were adapted to the conditions of determination of mechanical and optical properties but had no aesthetic significance. The 3D printing was performed on a heated (60 °C) silicon substrate to make removing the printed pattern from the surface easier. The G-codes for both objects were extracted in MatterControl, and the printing conditions were adjusted during the actual 3D printing test. Greater *z*-axis adjustment, print speed, and surface coverage of the printed object were needed. In Table 2, a basic summary of the printing conditions is presented. The printhead temperature was set to a minimum value, allowing the 3D printer to work. It was also crucial for this study that all objects printed with all three hydrogel samples were printed under the same conditions, thus achieving comparability of the results and neutralizing the influence of the printing conditions on the final quality of the printed foils. Three-dimensional-printed foils were air-dried at room temperature for six hours after printing. This drying method may not be optimal for packaging materials, but we found it suitable for our initial experiments. The prolonged drying time was manageable for thin foils, and since no additional energy was consumed for drying, it did not pose a significant issue. However, we recognize the need to explore more efficient drying methods for future research, particularly when printing thicker elements.

### 2.5. Analysis of 3D-Printed Foil Properties

#### 2.5.1. Morphology Study

After printing, samples were visually compared, photographed, and evaluated.

#### 2.5.2. Scanning Electron Microscopy (SEM) Analysis

The surface of 3D-printed foils was examined using SEM microscopy (JSM 6060 LV, JEOL, Tokyo, Japan). Small pieces of foil were cut out and stuck onto the SEM sample holder. A thin layer of gold was applied to them to reflect the surface area of each sample. The sample holders were then transferred to a microscope, the individual samples were examined, and images were captured at different magnifications.

#### 2.5.3. Ultraviolet-Visible (UV-VIS) Analysis

UV-VIS analysis, or spectrophotometry, which considers the ultraviolet and visible parts of the electromagnetic spectrum to analyze substances, was used to determine the transparency of 3D-printed foils. A UV-VIS Lambda 850 spectrometer (PerkinElmer, Shelton, CT, USA) was used for the purpose of this research. Measurements were carried out between 200 and 800 nm wavelengths with a step of 2 nm.

#### 2.5.4. Characterization of Physical–Mechanical Properties

The basic properties include thickness, mass, and density. Thickness was measured using a universal micrometer (Frank—PTI, Birkenau, Germany) according to a standard procedure (SIST EN ISO 534:2012) [49]. The mass was determined from 10 individual samples of each of the three 1 × 2 cm foil variations, weighing each rectangle and then averaging them.

The density was calculated from the mass and thickness measurements obtained. The porosity of the sample was not taken into account.

The tensile properties were determined using a Z010 dynamometer (Zwick Roell, Ulm, Germany) according to ASTM D882 [50]. The measurements were carried out under the same conditions: 40 mm clamping distance, 25 mm/s jaw movement speed, 1.5 × 7 cm test sample size, and 8 measurements per sample.

#### 2.5.5. Surface Characteristics

Gloss was measured using the Rhopoint IQ 20/60/85 Gloss Haze DOI Meter (Rhopoint Instruments, Hastings, UK) in accordance with the ASTM D523-14(2018) [51] standard at 20° and 60°.

The water contact angle was measured using a FibroDat dynamic wetting angle meter (TQC Sheen, Metamora, MI, USA) according to ASTM D5946-17 [52]. The test samples were slightly trimmed and adhered to the test sites with double-sided adhesive tape, and then tested by adjusting the needle with water at a volume of 5 μL per sample. The device captured a large number of screenshots of the drop on the sample, and then the final contact angle of the samples was calculated based on the time and images.

## 3. Results and Discussion

### 3.1. Laboratory Production of Mixtures of Hydrogel and Cationic Starch

Three samples of bacterial nanocellulose hydrogel were produced as part of the research. All contained 2% finished dry matter by weight and differed in the individual ratios of bacterial nanocellulose to cationic starch, i.e., 70/30, 60/40, and 50/50, respectively.

### 3.2. Structural Analysis

After homogenization of the BNC and ultrasonic treatment, the BNC had an average fibril width of 45 nm with lengths up to several hundred nm, according to the previous SEM study [12]. The yield of BNC from the initial solution (mother of vinegar) was ~87%. The rest were removed as impurities (mainly brownish-colored particles of the apple pulp that had served as the raw material for the vinegar production).

**Figure 1:**
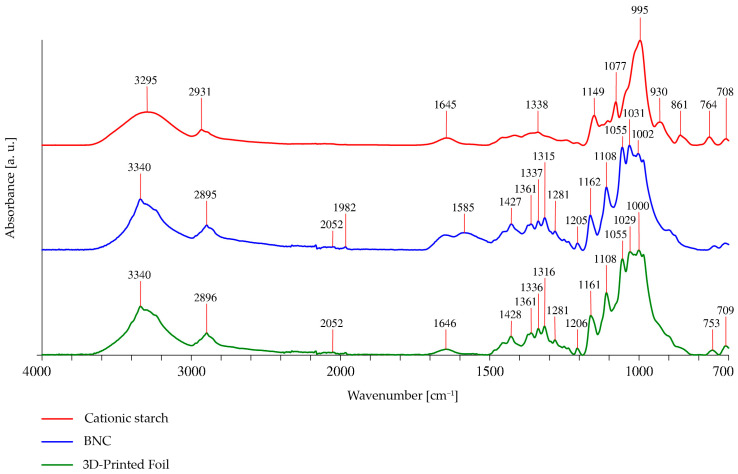
FT-IR spectra of BNC, cationic starch, and 3D-printed foil.

The crystallinity of the BNC was determined using XRD analysis. Its crystallinity amounted to 78%, with a zeta potential of −26.27 mV. The FT-IR spectra of BNC, cationic starch, and 3D-printed foil are presented in Figure 1. Because they are both polysaccharides, cellulose and cationic starch exhibit similar peaks in their FT-IR spectra. The peaks in the wave number range of 3660–2900 cm^−1^ are characteristic of the stretching vibration of O-H and C-H bonds in polysaccharides, and a strong, broad peak at 3340 cm^−1^ is assigned to different O-H stretching modes [53,54]. The peak at 2896 cm^−1^ is attributed to the CH stretching vibration of hydrocarbon constituents in polysaccharides [55]. In the region of 1644–900 cm^−1^, typical peaks that belong to stretching and bending vibrations of -CH_2_ and -CH, -OH, and C-O bonds in cellulose are present [53]. The peak located at 1644 cm^−1^ corresponds to the vibration of water molecules absorbed in polysaccharides. According to the literature [56], the stretching and deformation modes of quaternary ammonium functional groups are located at about 3100–3200 cm^−1^, 1485, 950, and 750 cm^−1^. FT-IR spectra show that in these regions, intense broad peaks are present, which hide the mentioned weak peaks (Figure 1). Pal et al. [21] assigned the small peak appearing at 1417 cm^−1^ to the stretching vibration of C-N in cationic starch. This peak is seen only in the spectra of cationic starch. Analysis of the spectra of 3D-printed foils did not reveal any significant differences between samples.

The interaction between the cationic starch and BNC involves multiple mechanisms, including electrostatic attraction, hydrogen bonding, and steric effects. Both NFC and cationic starch contain hydroxyl (-OH) groups in their molecular structures, which results in hydrogen bonding between both components. The hydrophilic nature of both materials facilitates the formation of hydrogen bonds with water molecules in hydrogels, which can further enhance interactions between BNC and cationic starch [57].

The zeta potential of the quaternary ammonium groups of cationic starch is typically positive, inducing an overall positive zeta potential at the surface of the cationic starch [58]. On the other hand, negatively charged groups of BNC give a relatively high negative zeta potential of −26.27 mV of BNC dispersed in the water. The positively charged surface of cationic starch can interact electrostatically with the negatively charged surface of BNC, stimulating adsorption and binding.

The degree of substitution (DS) in cationic starch affects its bonding ability with cellulose [59]. Higher DS values generally result in stronger and more stable interactions between both components because of increased hydrophilicity and a higher positive charge density on the starch surface, which enhances the electrostatic attraction between cationic starch and negatively charged cellulose. In our case, the DS value of cationic starch is low, in the range of 0.039–0.045, as reported by the producer, meaning that adsorption and the bonding ability are, besides electrostatic interactions, largely influenced by other parameters [60]. Cationic starch can exhibit a coil-like structure, and the degree of coiling determines the adsorption of the starch on the surface of cellulose. At low DS, the repulsion between the substituted segments is small, leading to a coiled conformation [60]. The coiled conformation can expose more binding sites on the starch molecule, enhancing its interaction with the cellulose surface and increasing its surface area coverage.

### 3.3. Rheological Analysis

#### 3.3.1. Oscillatory Amplitude Tests

**Figure 2:**
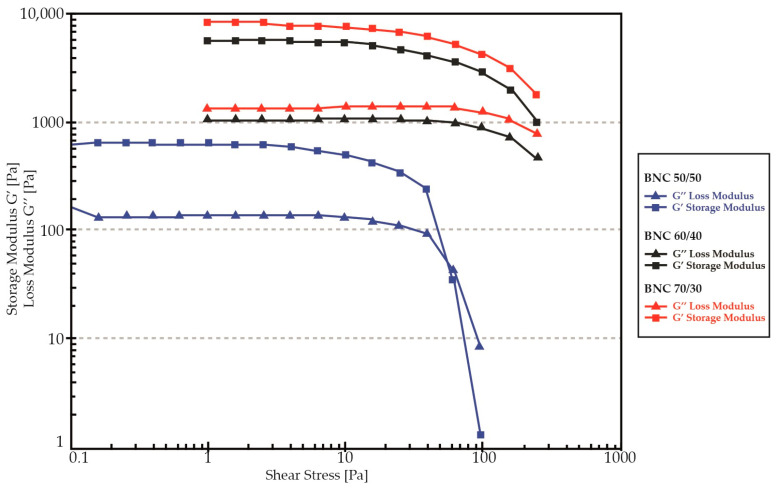
Oscillation amplitude tests at a constant oscillation frequency of 1 Hz (blue—BNC 50/50, black—BNC 60/40, red—BNC 70/30).

Viscoelastic materials exhibit both elastic and viscous behavior, where the storage modulus (G’) represents the energy elastically stored. In contrast, the loss modulus (G’’) represents the energy consumed as heat due to viscous effects. To determine the viscoelastic properties of the prepared gels, oscillatory amplitude tests were first performed at a constant oscillation frequency. These tests were also carried out to determine the range of linear viscoelastic responses in which the dynamic moduli (G’ and G’’) were constant and independent of the shear stress and strain. Figure 2 shows the results of amplitude tests on three hydrogels of bacterial nanocellulose. It can be seen that the dynamic modulus values for the BNC 60/40 and BNC 70/30 samples are comparable, while the modulus values for the BNC 50/50 sample are significantly lower. In all samples, it can be observed that in the low shear stress range, the storage modulus clearly dominates over the loss modulus, indicating a strong solid-like behavior. In this shear stress range, the values of the two moduli are constant, and then, at the critical shear stress point, the moduli start to decrease. At the critical shear stress, there is a transition from a linear to a non-linear viscoelastic response. As the shear stress is further increased, especially for the BNC 50/50 sample, a transition from a strong solid-like structure to a fluid-like structure can also be observed as the moduli equilibrate (G’ = G’’), and at higher shear stresses, the loss modulus starts to dominate, indicating the fluid character of the material in this stress range.

In general, it can be observed that for all samples, the storage modulus values are higher than the loss modulus values; this is indicative of the viscoelastic properties of the solid, which is a primary prerequisite for successful 3D printing [36]. In general, the BNC 70/30 sample has the highest values, the BNC 60/40 sample has slightly lower values, and both have about 1000 Pa higher loss modulus values and about 7000 Pa higher storage modulus values compared to the BNC 50/50 sample. It may be noted that the BNC 50/50 sample has the lowest linear response range of around 10 Pa, and consequently, its structure will fail at lower loads than the other two samples. This suggests that BNC 60/40 and BNC 70/30 could have been 3D-printed at much higher pressures than BNC 50/50, but due to the printer used in this study, these pressures were not reached, and thus the structure of neither sample was destroyed.

#### 3.3.2. Oscillation Frequency Tests

**Figure 3:**
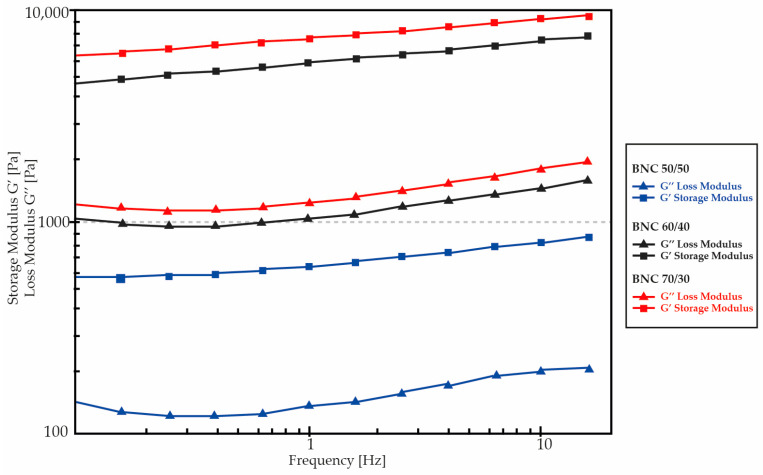
Oscillation frequency test—storage module and loss module vs. frequency (blue—BNC 50/50, black—BNC 60/40, red—BNC 70/30).

Figure 3 shows the results of frequency measurements of the BNC samples taken at a stress constant in the range of the linear viscoelastic response (previously determined by amplitude tests: Figure 2). Again, greater variation between the BNC 50/50 sample and the BNC 60/40 and BNC 70/30 samples can be observed, while the shape of the curve remains similar for all of them. Notably, the dynamic moduli (G’ and G”) show very little frequency dependence, with the storage modulus dominating over the entire range of frequencies studied; all samples exhibit gel-like behavior, as suggested by subsequent experimental work.

**Figure 4:**
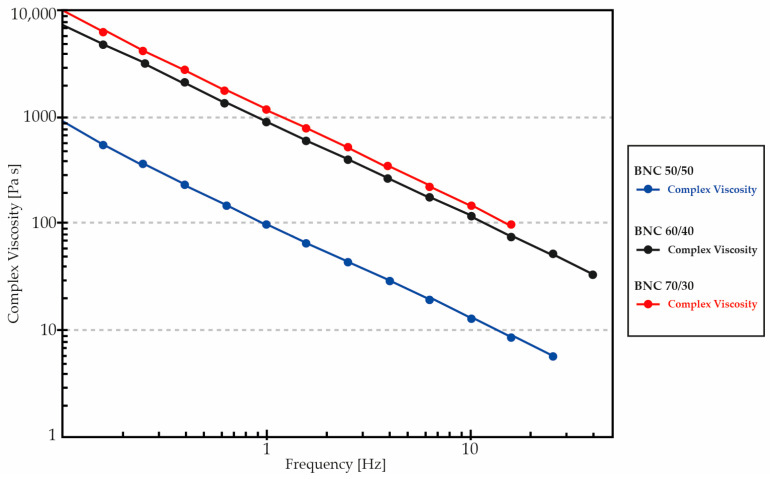
Frequency test, complex viscosity vs. frequency at 1 min pre-treatment γ͘ = 0.0001 s^−1^ (blue—BNC 50/50, black—BNC 60/40, red—BNC 70/30).

Figure 4 shows the dependence of the complex viscosity of the samples on the oscillation frequency. All three samples show a linear decrease in viscosity with increasing frequency in log–log coordinates. As the results of the previous graphs have already indicated, the BNC 70/30 sample shows the highest viscosity, the BNC 60/40 sample is only slightly less viscous than the latter, and the BNC 50/50 sample shows the lowest viscosity, i.e., around 900 Pa at the lowest frequency. It can, therefore, be assumed that no permanent deformation or spillage of hydrogel will occur in the treatment area up to approximately 25 Hz.

### 3.4. 3D Printing on a Prusa MKS3+ 3D Printer with FDM Technology

#### 3.4.1. Preparing the 3D Printer

For the purpose of this study, the Prusa i3 MKS3+ 3D printer (Prusa Research a.s., Prague, Czech Republic) was modified based on the research of Bessler et al. [45] and successfully adapted to the 3D printing of hydrogels with a few upgrades and modifications. The current capacity of the printer is somewhat limited, as the maximum amount of hydrogel that could be printed without interruption was 10 mL. According to our experiments, the content of one syringe was equivalent to 3D printing a 5 × 5 cm plate, but printing larger objects would require converting the printer to a continuous stream of hydrogel delivery.

The redesign of the Prusa 3D printer by Bessler et al. [45] has proven to be complex, given the updates to Prusa 3D printers since 2019, as both the software and some of the physical components of the printer have changed since then. Thus, problems in the assembly of 3D-printed parts were perceived, and solutions were found.

The software (PrusaSlicer 2.5.0) provided by the manufacturers for the use of the Prusa MKS3+ 3D printer was found to be inflexible and unadaptable to the printing conditions during the printing process. To this end, MatterControl (2.22.05) was used for the actual 3D printing and the preparation of the files for printing, allowing a large number of adjustments and changes to the printing conditions during the actual printing process, which was crucial in our case, where the printer parts were customized and not original. Prusa’s 3D printer configuration includes a PINDA smart sensor that allows the printer to quickly and easily adjust the position of the print head; however, in our case, as this sensor was slightly offset due to the printed parts, a little more adjustment and offsetting of the print head were required for proper 3D printing.

#### 3.4.2. 3D Printing

**Figure 5:**
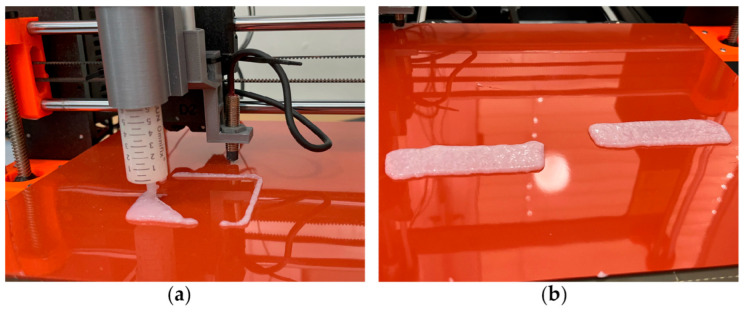
The 3D printing of the hydrogel with the Prusa MKS3+ printer. (**a**) The 3D printing process; (**b**) Final 3D-printed plates.

Throughout our research, we printed square and rectangular foils with all three hydrogels; the actual printing process and final 3D-printed plates can be seen in Figure 5. No major problems with the hydrogels were observed during 3D printing, and the 50/50 BNC foil was the smoothest to print, being the least dense and most homogeneous of the three. This proves the ability of materials, with the majority of their content being BNC, to be adequate for 3D printing—as most studies [39,40] conducted so far have utilized this form of nanocellulose only as an additive in very small quantities. Filling the syringes proved to be time-consuming, as it was necessary to manually unscrew the nozzle to the top of the structure during each filling, as the current design does not include lifting the syringe nozzle.

In further research, it would certainly be worthwhile to build on the patent and develop it to the point where it would allow continuous hydrogel delivery to the 3D printer, perhaps with a simple pump and a container holding a larger amount of material. In addition to this problem, the difficulty of filling the syringe with the denser of the three hydrogels was noted, as it was often the case that large amounts of air were drawn into the syringe, resulting in a poorer impression or even interruption of the print. There was also room for improvement in the material used to print the additional parts, as their opacity did not allow a view of the syringe, which could lead to air bubbles being overlooked in the upper part of the syringe. In future projects, it would be sensible to 3D print the part where the syringe is embedded with a more transparent polymer to avoid this problem.

Despite the challenges described regarding the experimentation process, the preliminary 3D printing was successful. The process result was partially transparent, mechanically stable foil, ready for further research.

### 3.5. 3D-Printed Foil Properties

#### 3.5.1. Morphology Study

**Figure 6:**
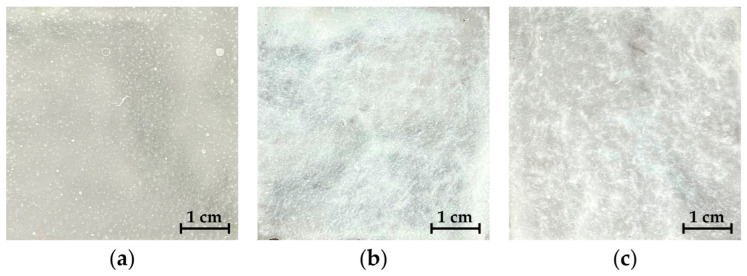
The 3D-printed foils, photographed on a black background. (**a**) BNC 50/50; (**b**) BNC 60/40; (**c**) BNC 70/30.

A visual comparison of the 3D-printed foils can be seen in Figure 6. All foils were evenly dried throughout the simple air-drying process and remained stable under the same conditions. This, compared to the study conducted by Leppiniemi et al. [34] with CNF-enriched hydrogels, showcases the ability of BNC to form and reduce the water content without the collapse of the structure. In our samples, there is some variation in the color of the samples, with samples B and C being slightly whiter but, at the same time, slightly less homogeneous than sample A. In terms of the waviness of the samples, it can be seen that foil A, the sample with the highest proportion of cationic starch, is the most uniform in plan, while sample C, the foil with the lowest proportion of cationic starch, is the waviest.

All samples are completely covered, with no holes or weak spots, while samples B and C show only a few areas of thinning and increased wrinkling of the bacterial nanocellulose (nano)fibrils.

#### 3.5.2. SEM Analysis

**Figure 7:**
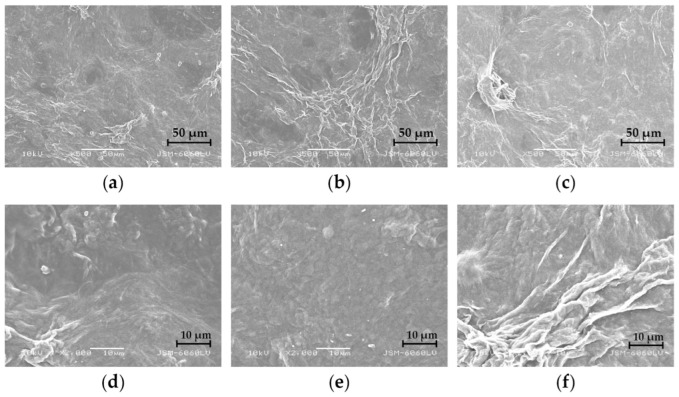
SEM images of BNC samples at 500- and 2000× magnification; (**a**) BNC 50/50, 500× magnification; (**b**) BNC 60/40, 500× magnification; (**c**) BNC 70/30, 500× magnification; (**d**) BNC 50/50, 2000× magnification; (**e**) BNC 60/40, 2000× magnification; (**f**) BNC 70/30, 2000× magnification.

Microscopic images of the samples were taken at 100, 500, 1000, 2000 and 10,000× magnification. A comparison of 500- and 2000× magnification images can be seen in Figure 7. A comparison of 100-, 500-, 2000- and 10,000× can be seen in Supplementary Materials, Figures S1–S3.

In the SEM images (Figure 7), all three 3D-printed foils look quite similar. In all of them, there is complete coverage of the surface, with no visible holes, defects, or areas without fibrils, which means that the fibrils have been evenly bonded with the cationic starch. Darker or slightly thinner areas can be seen in all three 3D-printed foils, the least of which can be seen in the BNC 70/30 foil. When examining a larger area of each sample, there are wrinkled areas, the least in the BNC 50/50 sample and the most in the BNC 70/30 sample. This suggests that the higher BNC concentration resulted in more wrinkled and less uniform areas, which can also be confirmed by visual assessment, as after drying, the foils with the higher BNC concentration took on a more twisted shape and were much less flat in appearance. The higher the amount of BNC in the sample, the more bubbles are observed in the foils, which is most likely a result of the density of the sample and, thus, the inability of air to escape from the printed hydrogel.

#### 3.5.3. UV-VIS Analysis

**Figure 8:**
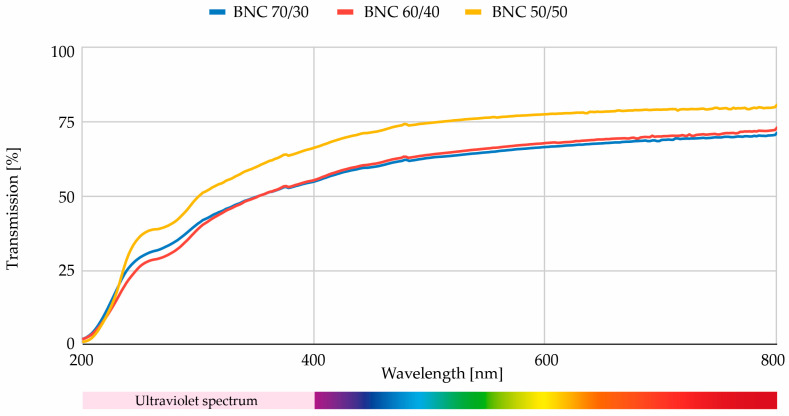
Transmission of 3D-printed foils (blue—BNC 70/30, red—BNC 60/40, yellow—BNC 50/50).

**Figure 9:**
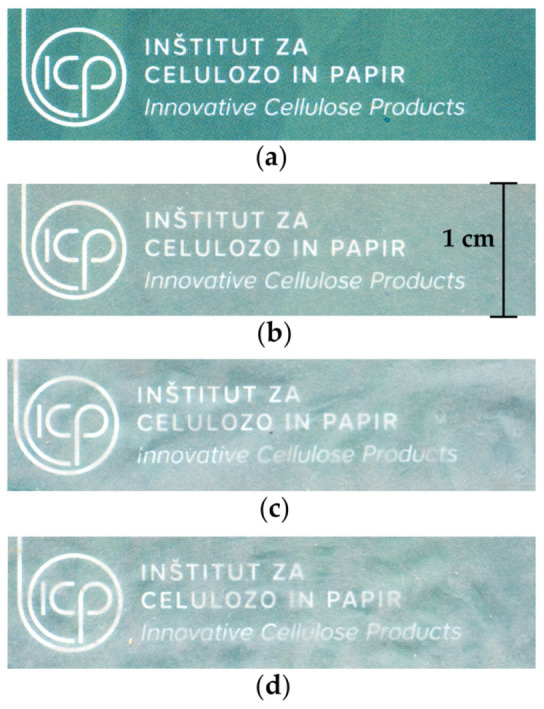
Visual representation of the semi-transparent quality of 3D-printed BNC foils; (**a**) Printed sample as a reference; (**b**) BNC 50/50 foil on top of the reference sample; (**c**) BNC 60/40 foil on top of the reference sample (**d**) BNC 70/30 foil on top of the reference sample.

Figure 8 shows a plot of the transmission results of three 3D-printed foils. It can be seen that the curves are similar for all three samples, and the transmission values of the BNC 50/50 foil are generally slightly higher than the other two. We see that in the ultraviolet part, all samples transmit less light. In the visible spectrum, the values increase slightly with wavelength. Overall, the BNC 50/50 foil transmitted the most light, i.e., about 10% more on average than the other two foils. This finding, seen in Figure 9, also confirms the initial observations, as these foils are also visually slightly more translucent. The differences between BNC 60/40 and BNC 70/30 are minimal, sometimes even overlapping. The graph does not show any major deviations, thus showing a more intense transition at specific wavelengths. Figure 9 shows scanned images of 3D-printed foils laid on top of a sample text to visualize their semi-transparent qualities.

#### 3.5.4. Characterization of Physical–Mechanical Properties

**Basic characteristics**

Table 3 shows the results of the measurements and calculations of the grammage, thickness, and density of the individual samples. Note that the grammage values are highest for the BNC 50/50 sample and lowest for the BNC 70/30 sample. Comparing the two results with the BNC 60/40 sample, there is a slight bias in the positive direction, but when the coefficient of variation is taken into account, it can be seen that the variation in the grammage values is relatively large for the BNC 60/40 sample, i.e., 16.7%, and slightly lower for the other two samples, i.e., around 6%, which makes any variation in the results of the BNC 60/40 sample comparable.

Given the expected linear variation in the other parameters, we can observe a small deviation between the results for density. The BNC 60/40 sample with the lower density value has a relatively larger measurement error, given the coefficient of variation calculated for the sample grammage measurements as well as for the density, which may require a more extensive range of measurements in the future or an adjustment of the testing conditions, which in this case were in accordance with the standard. These results also confirm our observations during the measurements, which were hampered by the waviness and spatial heterogeneity of the individual samples. In general, an increase in both grammage and density can be detected as the concentration of cationic starch in the samples increases, which can be attributed to the small size of the starch particles filling all possible spaces between the fibers of the bacterial nanocellulose.

We observed a slightly higher thickness value for the BNC 60/40 sample but also a slightly higher coefficient of variation. The thinnest sample was BNC 50/50, which is consistent with the density and grammage data, given that all samples were printed under the same 3D printer conditions.

**Tensile properties**

Table 4 shows the results of the tensile properties measurements of the foils as the mean of the eight sample measurements and their standard deviation.

As can be seen from Table 4, the tensile properties of the different 3D-printed foils vary considerably. The elastic modulus varies, with values between 2210 MPa for BNC 50/50 and 240 MPa for BNC 70/30. This shows that the sample with the highest concentration of cationic starch is able to withstand the highest stresses without significant deformation because the material is relatively stiff. The sample with the lowest amount of cationic starch is the most sensitive, with breakage occurring at around one-tenth of the BNC 50/50 sample value. Thus, a strong influence of the amount of cationic starch on the elastic modulus of all 3D-printed foils is evident.

According to the results, the elastic limit, or yield strength, also depends on the amount of cationic starch in the samples. Thus, the highest values of yield strain were achieved by the sample with the least cationic starch, i.e., BNC 70/30, at around 1.3%, reaching a yield strength of around 20 MPa. In contrast, the lowest values were achieved by the sample with the highest concentration of cationic starch, i.e., BNC 50/50, with an elongation of 0.9%, reaching a yield stress of around 40 MPa. Thus, the results confirm the strength and rigidity of the latter sample.

There is a slight variation in the tensile strength results, which the coefficient of variation can partly explain. As expected, the minimum tensile strength is indicated by the BNC 70/30 sample, while the maximum is indicated by the BNC 60/40 sample. Thus, the weakest of the 3D-printed foils requires about 29 MPa to break, whereas the stronger is almost twice as much, 51 MPa. Notably, the standard deviation values for both are 8 and 10 MPa, where some overlap may occur, thus achieving the same values or values consistent with the rest of the results.

The other results can be confirmed based on the measured strain at the break. The foil with the highest amount of cationic starch had the lowest elongation, being the most rigid of the three, and the foil with the lowest proportion of cationic starch had the highest elongation. Thus, the BNC 50/50 sample was stretched by only 1.2% at break and the BNC 70/30 sample by 1.9%.

According to the results, the BNC 60/40 sample required the most work to break, while the BNC 70/30 sample required the least. Again, the tolerance values are slightly higher, so a range of overlapping values is possible, but 7 mJ was still required to break the weakest of the foils.

The results of the energy density required for rupture show a similar pattern of values. This value is highest for the BNC 60/40 specimen, at approximately 274 kJ/m^3^, and lowest for the BNC 70/30 sample, at around 190 kJ/m^3^. Materials with a higher tensile energy density can withstand greater tensile forces before breaking and are considered tougher and more resilient.

**Figure 10:**
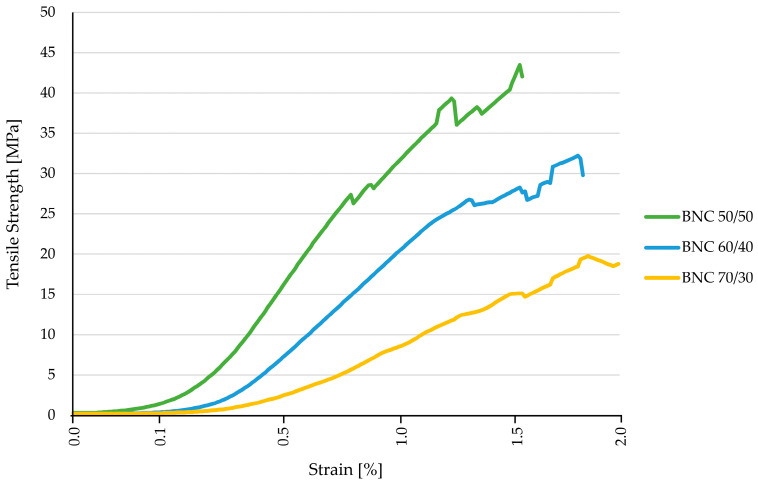
Graphical representation of elongation versus strength of 3D-printed foils.

In general, it is evident that 3D-printed foils, especially BNC 50/50, exhibit significant stiffness while concurrently demonstrating relatively high rupture forces. This trend is also visually apparent in Figure 10. Highly crystalline BNC has increased stiffness and rigidity, which results in a higher elastic modulus and tensile strength. The rigidity of BNC and the formation of a stiff percolating network linked by hydrogen bonds and entanglements also reduce the strain at the break of foils.

From a transparency standpoint, commercial fossil-based polymers tend to be more transparent, which can be advantageous in certain cases as it allows users to see the packaged product clearly. Given that tensile properties are fundamental criteria for packaging materials, the results of measured tensile strength and strain at break of all foils were of utmost importance. Various foil compositions yielded significantly different mechanical behavior, a critical aspect for maintaining the foil’s authenticity and ensuring it withstands environmental impacts during packaging. Tensile strength indicates the maximum load per cross-sectional area the film can bear, while strain at break determines the extent of stretching the film can undergo before rupturing. The type of polymer, material composition, manufacturing process, thickness, and environmental conditions influence the tensile properties of foils.

Three-dimensional-printed hydrogels obtained with stereolithography, extrusion, or ink-jet printing differ from low-strength soft materials to tougher, stronger materials. The tensile strength of nanocellulose-based 3D-printed hydrogels can range from a few kPa, as reported in [40], to several MPa, as reported in [61]. Similarly, the elastic modulus shows much lower values from 1 to 35 MPa [40,61]. The reported data are much lower than the tensile properties of our samples (Table 4).

The tensile properties of 3D-printed foils compared to most commonly used synthetic packaging foils are lower, in the same range, or even higher. In Table 5, the tensile strength, elastic modulus, and strain at break of synthetic foils [62] are shown. The tensile strength and elastic modulus of 3D-printed foils, especially the BNC 50/50 sample, are comparable to most commercially used synthetic foils, whereas strain at break is much lower (Table 5). Compared to bio-based foils, 3D-printed foils exhibit similar or even better tensile properties. PLA with a tensile strength of 26–52 MPa, an elastic modulus of 2.6 GPa, and a strain at break of 4.7% [63,64] has comparable properties to BNC 50/50, whereas the tensile strength of cellophane with 25 MPa is lower and the strain at the break has a value around 30% higher [65]. An important effect on the tensile strength is the uniform distribution of both components in the composite hydrogel. Li et al. [66] showed that the tensile strength of starch films was improved when NC was uniformly distributed in the starch matrix.

**Surface characteristics**

*Gloss*

Table 6 shows the results of the gloss measurements in the gloss unit. A first look at the results shows that all the values are low. Again, the waviness of the samples made the measurements more difficult, resulting in slightly higher bias values for some measurements. Gloss is influenced not only by the composition of the material but also by its surface structure. A smoother surface typically results in a higher gloss due to more uniform light reflection, whereas a rougher surface may create a microtextured surface that diminishes gloss by diffusing light unevenly. From the results shown below, the 3D-printed foils would be classified as non-gloss surface materials. Among the samples themselves, the BNC 50/50 sample, i.e., the sample with the highest cationic starch content, expectedly showed the highest gloss values. In contrast, the sample with the lowest cationic starch content, i.e., BNC 70/30, showed the lowest gloss values. The highest gloss value was measured for the BNC 50/50 sample at an angle of 60°, with a value of 5.2. In the context of our study, we expected the sample with the highest cationic starch content to exhibit superior gloss due to the smaller molecular size of starch molecules, allowing for a more even distribution and the formation of a smoother film compared to the relatively larger solid particles of bacterial nanocellulose.

*Contact angle*

Table 7 shows the results of the water contact angle measurements on the individual samples. The measurement itself was relatively difficult, as it was difficult to ensure a perfectly flat sample surface, which was necessary for successful measurement. Overall, the results are relatively high, with samples showing poorer wettability and water retention, mainly due to the roughness of the surface. The contact angle is highest for the sample with the highest cationic starch content, i.e., 83°, and lowest for the sample with the lowest cationic starch content, i.e., BNC 70/30, for which the value was approx. 66°. In all measurements, but most notably in the samples with higher cationic starch contents, partial dissolution of the sample was observed, as was the turbidity of the droplet on the sample, indicating the hydrophilicity of the samples. The stability was relatively good, as the shape of the droplets did not change significantly even after measuring 5 s. The measurements showed slightly poorer results in the case of slightly thinner areas of the sample, implying higher wettability in the case of thinner foils. As contact angle value is influenced by surface roughness, surface chemistry, and environmental conditions, higher values are probably the consequence of surface roughness. The bulky quaternary ammonium groups of cationic starch may sterically hinder the access of water molecules to the surface of bacterial nanocellulose, which could also explain higher contact angles at foils with higher starch content.

Understanding the interaction between foils and water is vital for packaging purposes. The hydrophobic or hydrophilic nature is often determined by surface-free energy and morphology. The contact angle of the surface with water is a crucial parameter for characterizing a material, providing insights into its absorption and adhesion tendencies. A lower contact angle indicates higher hydrophilicity and hygroscopicity of the foils. Packaging foils are often made from hydrophobic materials, such as PP and PE, with contact angles with water larger than 90° [68,69], as well as hydrophilic materials, such as PET, with contact angles around 75° [69,70]. Cellophane exhibits good wettability for aqueous solutions and polar solvents due to its relatively hydrophilic nature. For cellophane foil, contact angles with water are reported to be very low, in the range of 20 to 40 [71].

In general, all samples exhibit a rather rigid structure with relatively small elongations. This property could be improved by using an appropriate plasticizer. Correspondingly, high contact angles determined with water in the range of PET suggest promising prospects for potential future use of such foils, even at higher Technology Readiness Levels (TRLs).

## 4. Conclusions

In this study, rheologically relevant hydrogels with the prevailing content of bacterial nanocellulose were produced, differing in the amount of cationic starch added. The manufacturing process involved several steps. The bacterial nanocellulose samples were treated with a homogenizer and an ultrasonicator, filtered, and then mixed with cationic starch and water to the final form. This process was adapted to obtain optimized samples with the best possible homogeneity and indicate good printability. However, the process is time-consuming and should, therefore, be further researched and adapted.

Rheological studies confirmed the viscoelastic structure of the hydrogels, with the hydrogels with higher concentrations of BNC indicating higher consistency, i.e., higher dynamic moduli and complex viscosity.

The Prusa 3D printer (Prusa i3 MK3S+) was successfully adapted, and the process was further optimized, allowing the user to create their own economical printer in just a few steps without the need for major interventions in the mechanical parts or the software of the 3D printer. In the actual 3D printing with hydrogel, there was a perceived problem with the material feed, as the current formulation was adapted to a 10 mL syringe, which was just enough to print a maximum size of a 5 × 5 cm plated 3D object. However, the printer could be further upgraded, and constant hydrogel feed technology could be developed, possibly using a peristaltic pump.

The 3D-printed foils were visually compliant and mechanically stable at room temperature. The extruded material solidified evenly without uneven drying, maintaining a consistently connected and uniform surface. The 3D-printed foils with a higher amount of bacterial nanocellulose were slightly less transparent, significantly wavier, and showed slightly poorer tensile properties, whereas the 3D-printed foil with the highest amount of cationic starch was homogeneous, more transparent, and, despite its rigidity, very strong. The better properties of BNC 50/50 could be connected to the more homogeneous distribution of BNC and starch in the composite hydrogel. Based on all the test results obtained, this foil (BNC 50/50) is the most suitable for further research and optimization. Should the application of such foils require it, the addition of other natural materials to increase the elasticity of the material would be a good idea to consider. The 3D-printed foils have some disadvantages compared to cellophane foils, which are known for their flexibility, high clarity and transparency, and moisture resistance. On the other hand, the production of cellophane foils is an energy-intensive process that generates waste in the form of by-products and requires chemicals that can have an environmental impact if not managed properly. Besides using fewer chemicals and producing less waste, the main advantage of producing 3D-printed foils from BNC is using renewable feedstocks such as agro-industrial waste materials and several end-of-life scenarios; for example, they can enter the paper recycling process without influencing the recyclability of the paper. The 3D-printed foils are sustainable and align with the principles of the circular economy by promoting sustainable material use, resource efficiency, and waste reduction.

Though many studies deal with 3D-printable nanocellulose-based hydrogels, our research is innovative in many ways. We have used BNC obtained from the mother of vinegar for the preparation of hydrogels. Hydrogels were prepared only from two components, with the prevailing content of BNC, not just with the addition of nanocellulose to other components (in small amounts), and without using a cross-linking agent. Three-dimensional printing was performed on an altered commercial FDM device, resulting in obtaining semi-transparent mechanically stable 3D-printed foil with tensile properties that are comparable to commercial bio-based packaging foils.

The results are a stepping stone for further research, tailoring the foils to the application, and possible solutions in the packaging field.

## Figures and Tables

**Table 1:** Manufactured hydrogels by ingredients.

Sample	Total Dry Matter by Weight [%]	BNC/Starch Ratio
Hydrogel 1	2	50/50
Hydrogel 2	2	60/40
Hydrogel 3	2	70/30

**Table 2:** Summary of 3D foil printing conditions.

Temperature of the printing table	60 °C
Border printing speed	35 mm/s
Layer printing speed	20 mm/s
Occupancy rate of the printed object	20%
Nozzle diameter	1 mm
Offset in *z*-axis	1.62 mm

**Table 3:** The grammage, thickness, and density of the 3D-printed foils.

Sample	Grammage [g/m^2^]	Thickness [μm]	Density [kg/m^3^]
BNC 50/50	63 ± 4	80 ± 9	793 ± 44
BNC 60/40	59 ± 10	101 ± 21	583 ± 97
BNC 70/30	57 ± 4	91 ± 17	624 ± 42

**Table 4:** Tensile properties of 3D-printed foils.

Sample	Elastic Modulus [MPa]	Yield Strain [%]	Yield Strength [MPa]	Tensile Strength [MPa]	Strain at Break [%]	Work at Break [mJ]	Energy Density [kJ/m^3^]
BNC 50/50	2109 ± 1471	0.9 ± 0.5	40 ± 7	45 ± 8	1.2 ± 0.3	11 ± 3	206 ± 80
BNC 60/40	901 ± 640	1.7 ± 0.6	34 ± 8	51 ± 10	1.5 ± 0.2	12 ± 2	274 ± 123
BNC 70/30	237 ± 159	1.3 ± 0.3	20 ± 9	29 ± 10	1.9 ± 0.6	7 ± 2	190 ± 95

**Table 5:** Typical tensile properties of synthetic foils according to the literature [62,67].

Sample	Elastic Modulus [GPa]	Tensile Strength [MPa]	Strain at Break [%]
LDPE	0.2–0.5	8–31	100–965
HDPE	0.6–1.1	17–45	10–1200
PP	1.1–1.5	31–43	500–650
BOPP	1.7–2.4	120–240	30–150
PET	2.8–4.1	48–72	30–3000
BOPET	3.8	220–270	70–110
PVC	to 4.1	10–55	14–450

**Table 6:** Gloss of samples.

Sample—Measurement Angle	Gloss Unit [/]
BNC 50/50—20°	1.8 ± 0.1
BNC 50/50—60°	5.2 ± 1.3
BNC 60/40—20°	1.4 ± 0.1
BNC 60/40—60°	2.7 ± 0.6
BNC 70/30—20°	1.4 ± 0.1
BNC 70/30—60°	2.5 ± 0.6

**Table 7:** Water contact angle and droplet volume.

Sample—Time	Contact Angle [°]	Drop Volume [µL]
50/50—1 s	83 ± 12	6 ± 4
50/50—5 s	81 ± 18	7 ± 4
60/40—1 s	72 ± 19	2 ± 1
60/40—5 s	72 ± 20	2 ± 1
70/30—1 s	70 ± 7	2 ± 0
70/30—5 s	66 ± 7	2 ± 1

## Data Availability

Data are contained within the article and Supplementary Materials.

## Supplementary Materials

The following supporting information can be downloaded at: https://www.mdpi.com/article/10.3390/polym16111527/s1, Table S1: Example of calculations of dry matter and the amount of cationic starch required; Figure S1: SEM images of the BNC 50/50 sample taken at 100- (**a**), 500- (**b**), 2000- (**c**) and 10,000× (**d**) magnification; Figure S2: SEM images of the BNC 60/40 sample taken at 100- (**a**), 500- (**b**), 2000- (**c**) and 10,000× (**d**) magnification; Figure S3: SEM images of the BNC 70/30 sample taken at 100- (**a**), 500- (**b**), 2000- (**c**) and 10,000× (**d**) magnification; Product information for the cationic starch.
