# Supplementary material for: The Development of a Bacterial Nanocellulose/Cationic Starch Hydrogel for the Production of Sustainable 3D-Printed Packaging Foils
Source: Polymers (Basel). 2024 May 29;16(11):1527. doi: 10.3390/polym16111527 (PMC11174455; doi:10.3390/polym16111527)
Supplement: Supplementary file 1 [file polymers-16-01527-s001.zip › polymers-2876131-supplementary.pdf]

polymers

MDPI

Supplementary Materials

The Development of a Bacterial Nanocellulose/Cationic Starch Hydrogel for the Production of Sustainable 3D-Printed Packaging Foils

Špela Dermol, Bojan Borin, Diana Gregor-Svetec, Lidija Slemenik Perše and Gregor Lavrič

The following materials are available online.

Table S1: Example of calculations of dry matter and the amount of cationic starch required.

	before drying		after drying		control drying	
time (from - to)	10:43		10:43-11:43		11:48-12:03	
Temperature [°C]	105					
Date 13.4.2023	m0 (weight) [g]	37.8345				
	m0(beaker+sample1) [g]	39.1318	m1(beaker+sample1) [g]	37.9918	m2 (beaker+sample1) [g]	37.9918
	m0(sample1) [g]	1.2973	m1(sample1) [g]	0.1573	m2(sample1) [g]	0.1573
					Water content [%]	87.8748
					Dry matter [%]	12.1252

BNC sample mass [g]	19.25	Final mass of hydrogel BNC+starch [g]	194.53					BNC/starch 60/40, 2% concentration
		Dry matter in BNC sample [g]	Total mass of required dry matter for 2% concentration [g]	Dry matter in starch [g]	**Mixed mass of starch + water [g]**	**Starch concentration [%]**	TEST: concentration obtained [%]	
Desired concentration [%]	2.00	2.33	3.89	1.56	**175.27**	**0.89**	2.00	
	60% of 2% [%]	40% of 2% [%]				0.89		
	1.20	0.80						

BNC sample mass [g]	19.25	Final mass of hydrogel BNC+starch [g]	233.43					BNC/starch 50/50, 2% concentration
		Dry matter in BNC sample [g]	Total mass of required dry matter for 2% concentration [g]	Dry matter in starch [g]	**Mixed mass of starch + water [g]**	**Starch concentration [%]**	TEST: concentration obtained [%]	
Desired concentration [%]	2.00	2.33	4.67	2.33	**214.18**	**1.09**	2.00	
	50% of 2% [%]	50% of 2% [%]				1.09		
	1.00	1.00						

BNC sample mass [g]	19.25	Final mass of hydrogel BNC+starch [g]	166.74					BNC/starch 70/30, 2% concentration
		Dry matter in BNC sample [g]	Total mass of required dry matter for 2% concentration [g]	Dry matter in starch [g]	**Mixed mass of starch + water [g]**	**Starch concentration [%]**	TEST: concentration obtained [%]	
Desired concentration [%]	2.00	2.33	3.33	1.00	**147.49**	**0.68**	2.00	
	70% of 2% [%]	30% of 2% [%]				0.68		
	1.40	0.60						

(a) (b) (c) (d)

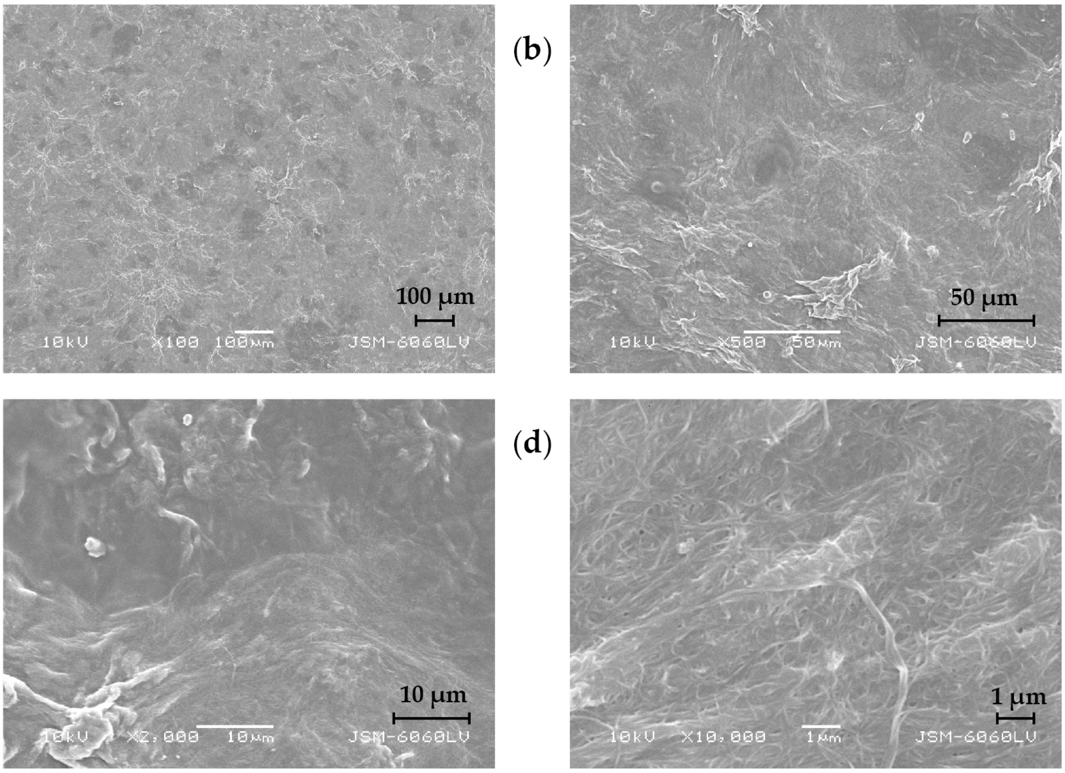

Figure S1: SEM images of the BNC 50/50 sample taken at 100- (**a**), 500- (**b**), 2000- (**c**) and 10000× (**d**) magnification.

(a) (b) (c) (d)

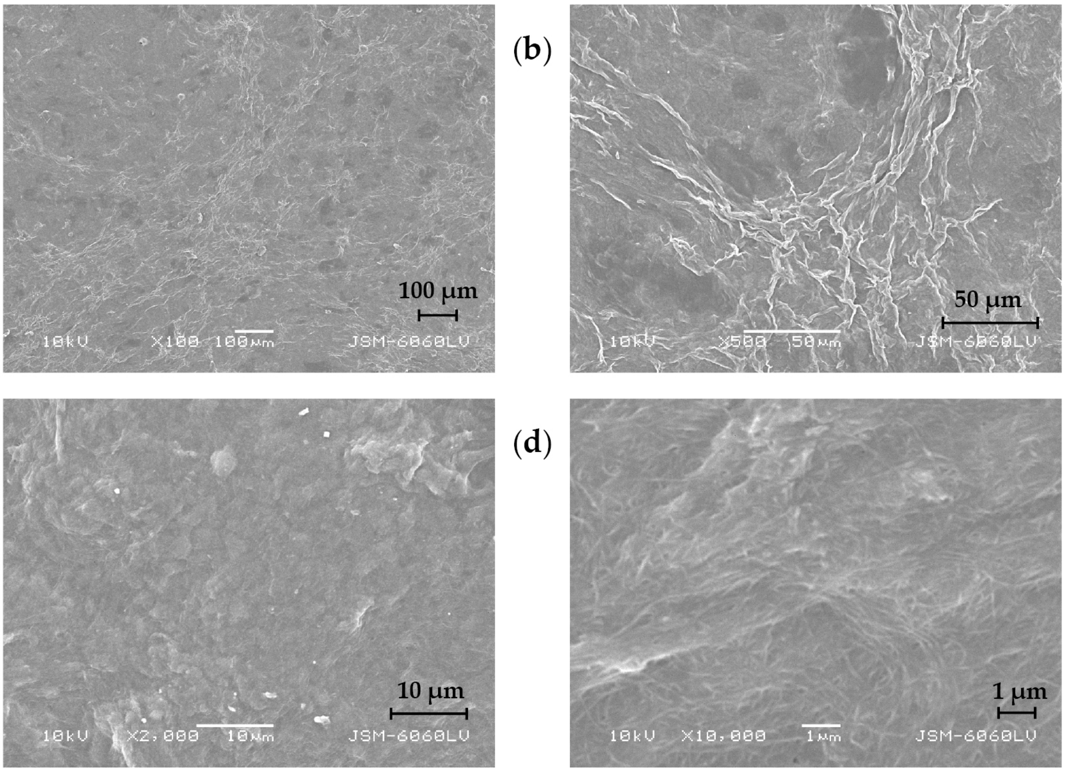

Figure S2: SEM images of the BNC 60/40 sample taken at 100- (**a**), 500- (**b**), 2000- (**c**) and 10000× (**d**) magnification.

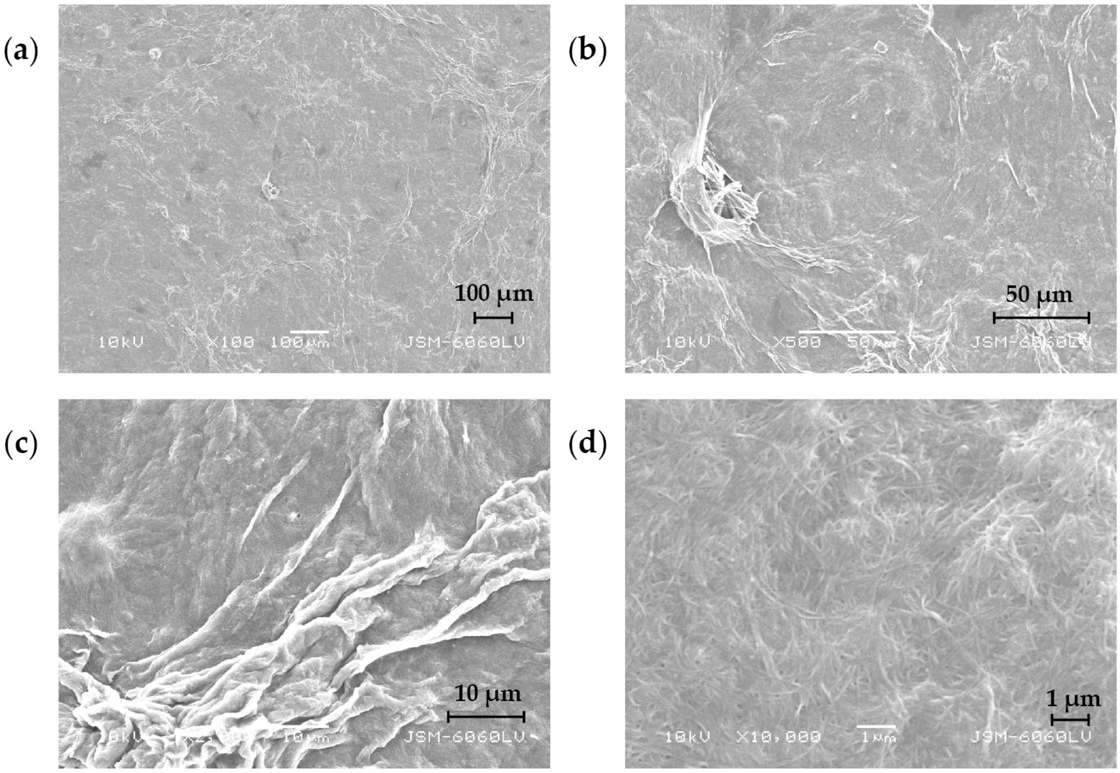

Figure S3: SEM images of the BNC 70/30 sample taken at 100- (**a**), 500- (**b**), 2000- (**c**) and 10000× (**d**) magnification.

PAPIRAN SKM 42

DESCRIPTION

This product is a medium D.S. cationic starch, available as a free flowing white powder.

PRODUCT LABEL

Package labelling Cationic starch

PRODUCT CLASSIFICATION

CN Code (Valid for EU 28) 3505 10 50

SPECIFICATIONS

Chemical/physical specifications

Parameter		Unit	Min	Typical	Max	Text
Moisture	-	%	10		13	
Cationic d.s.	-		0.039		0.045	
Flowability	cone height	mm	32		50	
pH slurry	20g + 100 ml		4		5.5	
Visc:Neutr RVA	6%ds,final	mPa.s	1200		1600	

Typical product data

Parameter		Unit	Typically
Bulk Density	loose	g/l	650
Bulk Density	packed	g/l	800

Legal requirements

ISO 14024 ss1 NORDIC ECOLABELLING for printing papers

BfR XXXVI, B.1.3 and C.IV.8 - Paper and board for food contact

BfR XXXVI/2 Paper and board for baking applications

This product is in compliance with:

FDA 21 CFR § 178.3520, Industrial starch-modified

This product is in compliance with GB9685:2016 Chinese National Food Safety Standard for Uses of Additives in Food Contact Materials and Their Products. According to GB 9685:2016 the product can be used with a maximum level of 1 % in paper and cardboard intended for food contact.

STANDARD PACKAGING

Bulk

Paper Bags

Big Bags

RECOMMENDED STORAGE CONDITIONS

Store inside, under dry conditions

FUNCTIONALITY

This product contains quaternary ammonium functional groups which confer a positive charge to the starch over a wide pH-range providing high starch self retention in paper stock systems.
It is not soluble in cold water.

APPLICATION

This product is used as a wet-end starch for the paper and board industry. It is not soluble in cold water.

Colloidal starch solutions are obtained by dispersion of the powder in water plus cooking by steam:
jet injection of steam: solids of the starch slurry up to 8% by utilizing fresh water (max 30°C), about 60 - 120 seconds cooking at 120°C-130°C (at 60 seconds cooking time - 130°C are recommended).
The paste concentration should be maximum 4-5 % in the storage tank and 0.5 % - 3 % when added to the pulp.
Typical starch addition is 0.7 % to 2.0 % calculated on dry pulp.

Prolonged storage of paste and slurry should be avoided as the starch does not contain any preservative.

Country of origin definition:

Country of Origin or product origin is defined as the country where the material was manufactured/produced/cultivated.
When the material undergoes substantial transformation in a second country, the country in which the transformation is performed shall be considered the country of origin.

Disclaimer:

This document is provided for your information and convenience only. All information, statements, recommendations and suggestions are believed to be true and accurate under local laws but are made without guarantee, express or implied. WE DISCLAIM, TO THE FULLEST EXTENT PERMITTED BY LAW, ALL WARRANTIES, EXPRESS OR IMPLIED, INCLUDING BUT NOT LIMITED TO WARRANTIES OF MERCHANTABILITY, FITNESS FOR A PARTICULAR PURPOSE and FREEDOM FROM INFRINGEMENT and disclaim all liability in connection with the storage, handling or use of our products or information, statements, recommendations and suggestions contained herein. All such risks are assumed by you/user. The labeling, substantiation and decision making relating to the regulatory approval status of, the labeling on and claims for your products is your responsibility. We recommend you consult regulatory and legal advisors familiar with applicable laws, rules and regulations prior to making regulatory, labeling or claims decisions for your products. The information, statements, recommendations and suggestions contained herein are subject to change without notice.
